# Descemet Membrane Endothelial Keratoplasty Graft Preparation Using the Liquid Bubble Technique with Subtrabecular Hydrodissection: A Retrospective Real-Life Study

**DOI:** 10.3390/jcm13206048

**Published:** 2024-10-10

**Authors:** Emmanouil Blavakis, Mateusz Kecik, Georgios D. Panos, Gabriele Thumann, Horace Massa

**Affiliations:** 1 Department of Ophthalmology, Geneva University Hospitals, 1205 Geneva, Switzerland; 2 Faculty of Medicine, University of Geneva (UNIGE), 1205 Geneva, Switzerland; 3 Department of Ophthalmology, Queen’s Medical Centre, Nottingham University Hospitals NHS Trust, Nottingham NG7 2UH, UK; 4 Division of Ophthalmology and Vision Sciences, School of Medicine, University of Nottingham, Nottingham NG7 2RD, UK

**Keywords:** Descemet membrane endothelial keratoplasty, graft preparation, liquid bubble technique, hydrodissection, time

## Abstract

**Background**: Techniques for the preparation of grafts for Descemet membrane endothelial keratoplasty (DMEK) can be classified into those that involve the manual dissection of the Descemet membrane (DM) and those that use an injection of a liquid or a gas to achieve a separation of the DM from the posterior corneal stroma. The purpose of this study was to evaluate the efficiency of the liquid bubble technique. **Methods**: The success rate of the technique was calculated retrospectively using the operating reports. Video files for each graft preparation were retrieved and the time, number of injections, and number of injections sites required for the hydrodissection were measured. The number of cases in which a manual dissection of the Descemet membrane was necessary was recorded. Information on donor age and graft preservation time were retrieved from the eye bank file. **Results**: In 58 cases, the success rate was 98.3%. In the 28 procedures where a video was available, the median time for hydrodissection was 4.4 min. The median number of injection sites was 2, with a median number of injections of 3.5. Manual dissection as a rescue technique was performed in 25% of cases, with one case resulting in graft tears. The mean graft diameter was 7.6 mm. The mean donor age was 66 years, and the mean graft storage time was 22 days. **Conclusions**: The liquid bubble technique can be a fast and valuable choice for DMEK graft preparation, especially in centers where the tissue is prepared in the operating theater.

## 1. Introduction

Descemet membrane endothelial keratoplasty (DMEK) was introduced in 2006 by Melles and has since become established for the treatment of corneal endothelial cell disorders such as Fuchs endothelial dystrophy and bullous keratopathy [1]. It consists of removing the patient’s Descemet membrane (DM) and replacing it with a thin layer of Descemet membrane that is dissected from a donor cornea along with its endothelial cells and then positioned onto the recipient’s cornea using an air or gas bubble [2].

Donor tissue preparation techniques can be classified into those that involve manual dissection of the DM and those that use an injection of a liquid or a gas to achieve a separation of the DM from the posterior corneal stroma [3]. In the different variations of manual dissection techniques, the DM is cut or dissected either anteriorly or posteriorly to the trabecular meshwork and then stripped using sterile forceps [1,4,5]. In pneumatic dissection, air is injected into the pre-DM plane, achieving separation of the DM and the posterior stroma [6]. Similarly, in hydrodissection, a liquid such as a culture medium, balance salt solution (BSS), or a vital dye can be injected into the pre-DM plane [3]. More specifically, in the no-touch liquid bubble technique, an injection site is created under the iris base and the Schlemm canal and a vital dye is injected into the pre-DM plane, resulting in the formation of a bubble. The DMEK lenticule is then harvested using a spatula after corneal trephination [7].

A study performed in an eye bank has shown that the preparation of a DMEK graft is faster using hydrodissection or pneumatic dissection techniques as they require less than half a minute, whereas manual dissection techniques may take from 8 to 20 min [8]. Manual dissection has also been associated with higher failure rates and higher chances of the formation of DM tears [9]. Few studies have assessed the success rate of hydrodissection techniques, and the time required for graft preparation when performed in the operating room.

The purpose of this study is to evaluate the liquid bubble technique with subtrabecular hydrodissection for the preparation of a DMEK graft in a real-life clinical setting.

## 2. Materials and Methods

This retrospective study was conducted in the Ophthalmology Department of Geneva University Hospitals. It adhered to national ethical regulations for clinical research and the Declaration of Helsinki and received a waiver for Ethics Committee approval by the Committee of Human Research of the Canton of Geneva.

**Figure 1:**
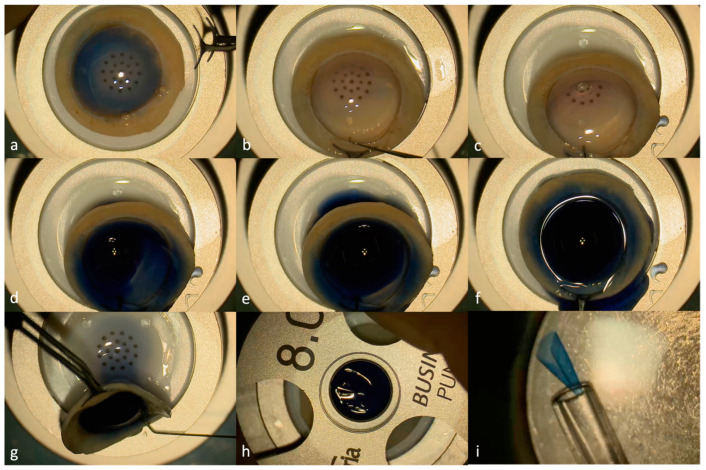
Steps of graft preparation using the liquid bubble technique: (**a**) donor cornea placed on a punch base with the endothelium facing up and stained with trypan blue; (**b**) creation of a narrow tunnel using a Sinskey hook under the trabecular meshwork and the Descemet membrane while the graft is held with sterile forceps; (**c**) the radial insertion of a 30G Rycroft anterior segment cannula connected to a syringe filled with balanced salt solution (BSS) and trypan blue into the stroma–Descemet membrane interface; (**d**) the trypan blue and BSS injection leading to bubble formation; (**e**) the complete formation of the bubble just before (**f**) and after removal of the cannula; (**g**) liquid removal by holding the graft vertically and by applying gentle pressure posteriorly to the injection site; (**h**) punch of 8 mm; and the (**i**) insertion of the graft into the injector prior to transplantation.

This study included all corneal endothelial grafts prepared for a DMEK, regardless of indication, using the hydrodissection technique from March 2018 to November 2023. There were no exclusion criteria. Graft preparation was performed by the same experienced surgeon (HM) under an operating microscope in the theater before the arrival of the patient (Figure 1).

The donor cornea was placed on a punch base with the endothelium facing upwards and was stained using trypan blue (Vision Blue^®^; DORC, Zuidland, The Netherlands). The donor tissue was held at the sclera using sterile forceps and no suction was applied. An area of about 1 mm in the periphery of the trabecular meshwork without any residual uveal tissue or previous corneal incisions was identified as a potential injection site. A narrow tunnel was created as the trabecular meshwork and the Descemet membrane were dissected locally by using a Sinskey hook and gently moving in a horizontal manner. A 30G Rycroft anterior segment cannula connected with a three-way valve to a syringe filled with BSS and trypan blue was then inserted radially into the stroma–Descemet membrane interface (i.e., pre-DM plane) without any occlusion or compression of the dissected orifice. Trypan blue with BSS was then injected by the assistant with enough pressure to separate the stroma from the Descemet membrane. When bubble formation was not possible after a few attempts, or when a small bubble that could not be expanded was created, a new injection site was chosen. This procedure was repeated until a bubble of satisfactory size was formed. The liquid was then removed by holding the graft vertically and by applying gentle pressure posteriorly to the injection site. A punch of 6.5 mm to 9.5 mm, depending on the needs of the intervention, was then used; the graft was harvested using a blunt spatula and was inserted in a bath of artificial aqueous humor. If separation of the Descemet membrane from the corneal stroma was not complete at that stage, manual dissection was used as a rescue technique and the DM was peeled manually using sterile forceps. The endothelial graft was then inserted into the injector before transplantation.

Successful graft preparation without the formation of any DM tears was evaluated using the operating reports and the success rate of the technique was calculated. Video files for each graft preparation were retrieved retrospectively from the operating theater’s database, and the time required for the hydrodissection was measured. This time was defined as the elapsed time between the beginning of the preparation of the first injection site and the formation of a satisfactory bubble. The number of injection sites created and the number of injections required were also recorded. In addition, the number of cases in which a manual dissection of the Descemet membrane was necessary as well as the number of cases where a tear of the Descemet membrane was created during manual dissection were measured. Finally, the size of the punch used for graft harvesting was documented.

Information about the age of donors as well as the time the graft was preserved in the eye bank were retrieved from the donor file of the eye bank. All data were recorded in a Microsoft Excel file (Microsoft 365, Microsoft Inc., Redmond, WA, USA).

## 3. Results

Out of 59 corneal endothelial grafts prepared for a DMEK using the liquid bubble technique with subtrabecular hydrodissection, a graft without any tears and suitable for transplantation was obtained in 58 cases (success rate 98.3%). In one case, manual dissection of the DM performed because of inadequate bubble formation led to the formation of multiple tears, rendering the graft unsuitable for transplantation. There were no other cases of DM tears.

**Figure 2:**
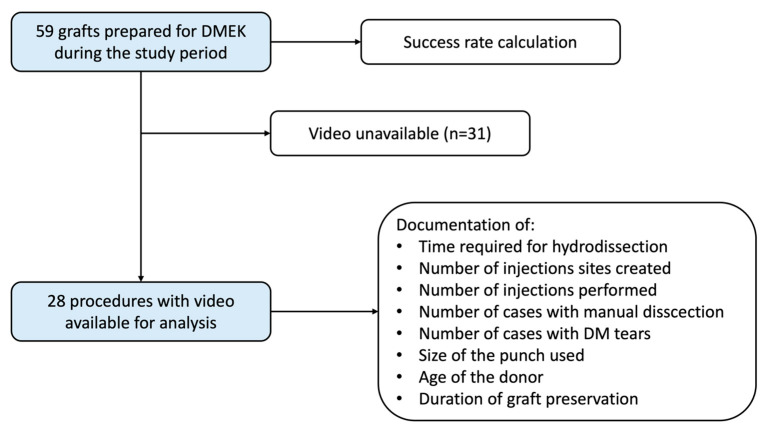
Flowchart.

**Figure 3:**
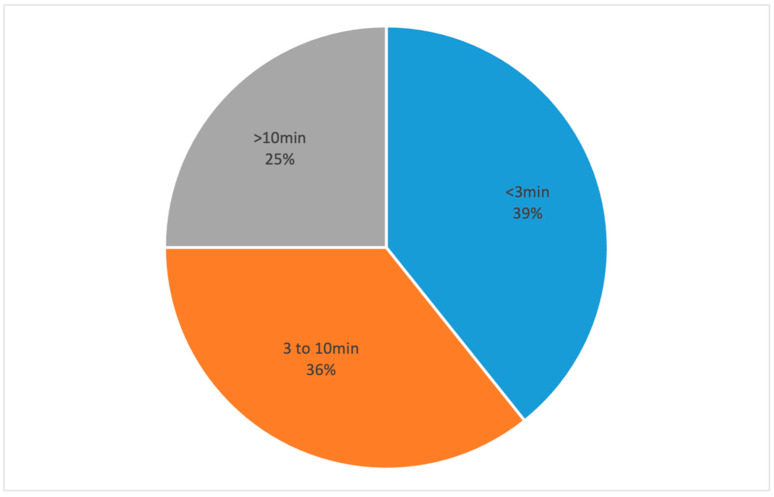
Pie chart showing the distribution of the time required for hydrodissection.

Video files were available for 28 procedures and they were included for further analysis (Figure 2). In those cases, the mean donor age was 66 (±13) years and the mean graft storage time was 22 (±7) days. The median time required for hydrodissection was 4 min and 25 s, whereas the mean time was 7 min and 53 s (±534 s). In 11 cases (39%), hydrodissection took less than 3 min, in 10 cases (36%) it was completed in a time frame from 3 to 10 min, and more than 10 min were required in seven cases (25%) (Figure 3). There was no correlation found between the time required for hydrodissection and donor age or graft storage time.

The median number of injection sites was 2 (2.9 injection sites on average) and the median number of injections required for a satisfactory hydrodissection was 3.5 (5.07 on average). Only 1 or 2 injections in 1 or 2 injection sites were required for the creation of a bubble of satisfactory size in 43% cases. In seven cases (25%), complete separation of the DM from the stroma was not achieved, leading to the utilization of the rescue technique of manual dissection. In that subgroup of the seven cases, the hydrodissection procedure lasted on average 13 min and 16 s (range 8.7–35.9 min) and required on average 10.4 injections (range 5–16) on four different injection sites (range 2–6).

**Figure 4:**
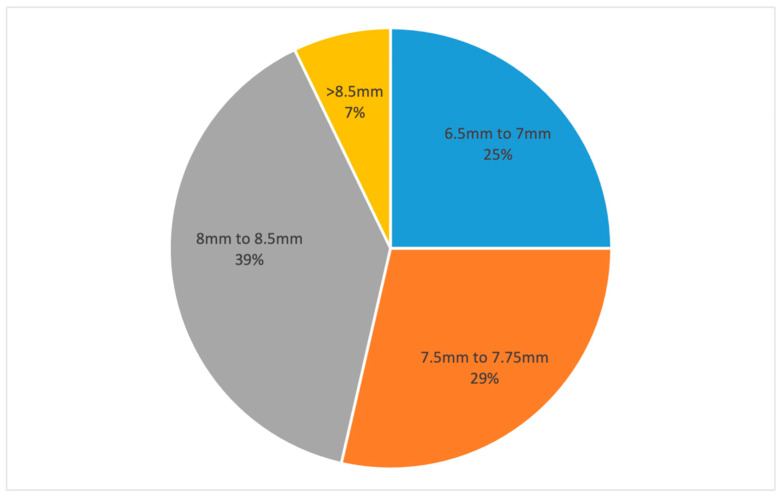
Pie chart showing the distribution of achieved graft diameter.

The mean graft size was 7.7 (±0.6) mm. A punch size of 6.5 mm to 7 mm was used in 25% of the procedures (n = 7), 7.5 mm to 7.75 mm in 28.6% (n = 8), 8 mm to 8.5 mm in 39.3% (n = 11), and more than 8.5 mm in 7.1% (n = 2) (Figure 4). Manual dissection of the DM was performed in 2 cases of a 7 mm punch, in 1 case of a 7.5 mm punch and in 4 cases of an 8 mm to 8.5 mm punch.

## 4. Discussion

This study aimed to provide insight into the details of a DMEK graft preparation using the liquid bubble technique with subtrabecular hydrodissection, focusing on the success rate, the time required for hydrodissection, and the number of injections and injection sites needed to achieve a liquid bubble of a satisfactory size.

In this study, the success rate of the technique was very high as it produced a graft suitable for transplantation in 98.3% of cases. In early studies of the hydrodissection technique, the success rate was lower, at 92% [10]. However, more recent studies report a success rate at 99%, comparable to that of this study [7]. The tissue loss rate due to failed preparation seems to be higher with manual dissection techniques and it has been reported to be as high as 24%, whereas studies performed later report a tissue loss rate that ranges from 1.3% to 8.5% [5,11,12]. These differences are to a great extent explained by the frequent formation of DM tears during manual dissection. In this study, there was a tear in the DM in only one case (3.6%) where manual dissection of the DM was performed as a rescue technique. There was no damage on the DM in cases where hydrodissection was complete and no rescue technique was required. In comparison, 1.5 to 5.9 radial tears per tissue prepared have been reported as occurring during the peeling of the DM in the studies of Sella et al. and Din et al. [9,13].

Despite numerous different techniques being available for DMEK lenticule preparation, only a few studies report the time required for graft harvesting [3]. In this study, in more than one third of the procedures, less than 3 min were required for the dissection of the DM and the mean time required for hydrodissection was 7.9 min. However, in one fourth of the cases, more than 10 min were needed. Similar studies report that about 3 min were necessary for DM hydrodissection [7,14], while in this study 61% of the cases required more than 3 min. Such differences could be attributed to the surgeon’s experience or to the fact that in our study the grafts were prepared in the operating theater before the surgery and not in the eye bank, requiring the surgeon to be more cautious in order to avoid any complications such as the tearing of the DM.

Hydrodissection and pneumatic dissection of the DM are generally considered to be faster than manual dissection of the DM, requiring a mean time of 0.17 min for pneumatic dissection and 0.27 min for hydrodissection versus from 7.6 to 19.4 min for the three different types of manual dissection of the DM in a comparative study by Parekh et al. [8]. The same study also confirmed that there is significantly more endothelial cell death with hydrodissection compared to manual peeling techniques and this is one of the main reasons that manual peeling is the method of choice for many surgeons [8]. This study further confirms that hydrodissection is faster than different manual dissection techniques that require 8.4 min in the modified SCUBA technique and from 11 to 16 min using other versions of manual dissection [9,13,15,16]. In certain cases, scoring and the dissection of the DM may require more than 20 min [17]. Pneumatic dissection has also been reported to require up to 20 to 30 min [12]. Hydrodissection and pneumatic dissection seem to be similar techniques, but hydrodissection is preferred by many surgeons because there is minimal or no residual stroma in DMEK grafts prepared with hydrodissection, whereas residual stroma can be found in all grafts prepared with pneumatic dissection [18].

The mean graft size in this study was 7.6 mm and most of the grafts were considered as grafts of standard diameter [19]. However, one quarter of the grafts obtained had a diameter of either 6.5 mm or 7 mm, which is relatively small. Even though large graft size is considered an advantage of the hydrodissection technique, the mean graft size of 11 mm achieved by other studies could not be reached in our study, where only 7.2% of the grafts had a diameter bigger than 8.5 mm [20,21]. The high percentage of donors with diabetes could explain the relatively small mean graft size as DMEK lenticules are thought to be more difficult to prepare from the corneas of patients with diabetes [17]. We managed to obtain grafts with a similar size to those produced by the “no touch liquid bubble technique” of Szurman et al. (8.3 mm) and comparable to most of the grafts retrieved with manual dissection techniques [3,7,19].

The main difficulty of the liquid bubble technique is the correct insertion of the needle into the pre-DM plane, which has led to the development of devices such as the DescePrep, which aims to facilitate this insertion [14]. This is also highlighted in our study where the mean number of injections required was 5.07 (median 3.5 and range 1–16), but in 43% of cases, one or two injections and injection sites were enough for bubble formation. This shows that the creation of an injection site can be challenging but if it is conducted correctly, bubble formation occurs rather effortlessly. The staining of the endothelial surface of the graft helps with the correct identification of the trabeculum. It is also of high importance to avoid areas of previous cataract surgery incisions when choosing the site of an injection as an incision in the DM anteriorly to the trabeculum can prohibit bubble formation.

When the formation of a liquid bubble of a satisfactory size is challenging, manual dissection can be used as a rescue method in hydrodissection techniques at any stage of the procedure. In this study, the manual dissection of the DM was necessary in seven cases (25%). Another known drawback of hydrodissection is the increased loss of endothelial cells that occurs compared to manual dissection and this is one of the main reasons that manual dissection is the method of choice in many corneal transplantation centers [3,8,22].

The difficulties in preparation of endothelial grafts have led to the development of several alternatives to keratoplasty for endothelial cell dysfunction [23]. The Descemet stripping only (DSO) technique with or without postoperative treatment with Rho-associated protein kinase inhibitors (ROCKi) has proved effective in certain cases [24,25]. Another broadly investigated alternative approach to endothelial keratoplasty is the injection of cultivated corneal endothelial cells (CEC) in the anterior chamber [26]. Techniques are also being developed for the transplantation of human CEC or CEC-like cells derived from stem cells through tissue engineered endothelial keratoplasty [27,28].

A major limitation of this study is that endothelial cell count data before harvesting and after transplantation were not available. Data regarding the history of cataract surgery or diabetes mellitus in the donor were also unavailable. Other limitations of this study include its small sample size and its retrospective character. The fact that it was conducted in a single center and grafts were prepared by a single surgeon means that results might also not be broadly applicable to diverse clinical settings and surgeons with different levels of experience. These limitations highlight the need for cautious interpretation of the results and suggest directions for future research.

To conclude, this study provides real-life data of DMEK lenticule preparation using a hydrodissection technique. It shows that hydrodissection can be faster than most of the manual dissection techniques and that in cases where satisfactory bubble formation is impossible, the manual dissection of the DM can be used effectively and with a low complication rate as a rescue method. Despite its drawbacks, the liquid bubble technique can be a valuable choice for DMEK graft preparation, especially in centers where the tissue is prepared in the operating theater where time is of high importance and efficacy of extreme value. Further studies are required in order to better compare hydrodissection with manual dissection techniques in various clinical settings.

## Data Availability

Data are available upon request.
